# Inhibition of PAR-1 delays aging via activating AMPK in *C. elegans*

**DOI:** 10.18632/aging.104180

**Published:** 2020-11-20

**Authors:** Di Wu, Waijiao Cai, Xuan Zhang, Jianfeng Lan, Lina Zou, Samuel J. Chen, Zixing Wu, Di Chen

**Affiliations:** 1 State Key Laboratory of Pharmaceutical Biotechnology, Model Animal Research Center of Medical School, Nanjing University, Pukou, Nanjing 210061, Jiangsu, China; 2 Institute of Traditional Chinese and Western Medicine, Huashan Hospital, Fudan University, Shanghai 200040, China; 3 Affiliated Hospital of Guilin Medical University, Guilin 541001, Guangxi, China; 4 Department of Mechanical, Aerospace, and Nuclear Engineering, Rensselaer Polytechnic Institute, Troy, NY 12180, USA

**Keywords:** longevity, healthspan, PAR-1, AMPK, *Caenorhabditis elegans*

## Abstract

The antagonistic pleiotropy theory of aging suggests that genes essential for growth and development are likely to modulate aging later in life. Previous studies in *C. elegans* demonstrate that inhibition of certain developmentally essential genes during adulthood leads to significant lifespan extension. PAR-1, a highly conserved serine/threonine kinase, functions as a key cellular polarity regulator during the embryonic development. However, the role of PAR-1 during adulthood remains unknown. Here we show that inhibition of *par-1* either by a temperature-sensitive mutant or by RNAi knockdown only during adulthood is sufficient to extend lifespan in *C. elegans*. Inhibition of *par-1* also improves healthspan, as indicated by increased stress resistance, enhanced proteotoxicity resistance, as well as reduced muscular function decline over time. Additionally, tissue-enriched RNAi knockdown analysis reveals that PAR-1 mainly functions in the epidermis to regulate lifespan. Further genetic epistatic and molecular studies demonstrate that the effect of *par-1* on lifespan requires the AMP-activated protein kinase (AMPK), and RNAi knockdown of *par-1* results in age-dependent AMPK activation and reduced lipid accumulation in the metabolic tissue. Taken together, our findings reveal a previously undescribed function of PAR-1 in adulthood, which will help to understand the molecular links between development and aging.

## INTRODUCTION

Numerous studies have demonstrated that aging can be modulated by evolutionarily conserved signaling pathways, among which the nutrient-regulated insulin/insulin-like signaling (IIS) and target of rapamycin (TOR) pathway have been shown to play a conserved role in aging in many species [1–3]. Mutations in *daf-2*, which encodes the IGF-1 receptor ortholog in *C. elegans*, more than double the adult lifespan [4, 5]. This significant lifespan extension is dependent on the downstream DAF-16/FOXO transcription factor [6, 7], which is completely or partially required for the anti-aging effect of many genetic or environmental manipulations. Inhibition of LET-363/TOR or its co-factor DAF-15/Raptor also significantly extends lifespan [8, 9]. The underlying mechanisms involve the ribosomal S6 kinase (S6K)-mediated regulation of mRNA translation, autophagy, lipid metabolism and so on [10–13]. Simultaneous inhibition of IIS and TOR pathway has a synergistic effect on longevity, suggesting these pathways actively interact with each other [14, 15].

Two genome-wide RNAi screens for increased longevity have been performed to systematically identify key modulators of aging in *C. elegans* [16, 17]. From more than 10,000 RNAi clones tested in each study, 89 and 23 genes were identified as negative regulators of longevity, respectively. However, there was only one gene identified in common from both studies, suggesting the screens are far from reaching saturation. During these screens, animals were treated with various RNAi throughout the whole life. Thus, essential genes, RNAi knockdown of which causes larval arrest, were unlikely to be tested for their effects on longevity. Later studies on essential genes by RNAi knockdown only during adulthood helped to identify more negative regulators of longevity [18, 19]. These essential genes tend to be evolutionarily conserved and are involved in basic biological processes, such as mRNA translation, mitochondrial functions, signal transduction and so on. Therefore, characterization of developmentally essential genes for their roles in lifespan and healthspan should shed light on the molecular link between development and aging.

Establishment of body axis polarization plays a critical role during the embryonic development. Six *par* genes (*par-1* thought *par-6*) regulate the anterior-posterior asymmetries during the first two embryonic cell divisions in *C. elegans* [20, 21]. PAR-1/MARK, a conserved serine-threonine kinase, plays a key role in establishing the cellular polarity [22]. During the first cell division, PAR-1 protein shows asymmetric distribution, which controls cytoplasmic and cytoskeletal asymmetries along the polarity axis. It was reported that PAR-1 also regulates vulval morphogenesis during larval development [23]. However, little is known about the biological functions of PAR-1 in adulthood.

In order to characterize the roles of PAR-1 in adult animals, we performed lifespan and healthspan assays and found that inhibition of *par-1* significantly delays aging. Spatiotemporal analyses reveal that *par-1* mainly functions in the epidermis during adulthood to regulate lifespan. Moreover, genetic epistasis studies demonstrate that the effect of *par-1* on longevity is independent of the insulin-like signaling. Instead, *par-1* functions through the nutrient-responsive S6K and AMPK to regulate lifespan. Inhibition of *par-1* results in age-dependent AMPK activation and reduced lipid accumulation in the metabolic tissue. Taken together, these results reveal previously undescribed roles of PAR-1 in aging and metabolism.

## RESULTS

### Inhibition of *par-1* during adulthood significantly extends lifespan

**Figure 1:**
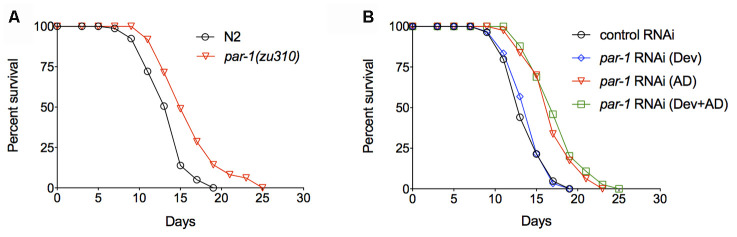
**Inhibition of *par-1* during adulthood is sufficient to significantly extend lifespan.** (**A**) Survival curves of the wild-type N2 and *par-1(zu310)* temperature sensitive mutant (*p* < 0.0001, log-rank test). (**B**) Survival curves of the wild-type N2 treated with either the control RNAi or *par-1* RNAi at different stages. Dev, *par-1* RNAi treatment during development (*p* = 0.5368, log-rank test). AD, *par-1* RNAi treatment during adulthood (*p* < 0.0001, log-rank test). Dev + AD, *par-1* RNAi treatment during the whole life (*p* < 0.0001, log-rank test). Detailed quantitative data and statistical analyses are included in Supplementary Table 1.

To characterize the functions of PAR-1 during adulthood, we took advantage of the *par-1(zu310)* temperature sensitive (ts) mutant [24] and examined the lifespan phenotype. Animals were cultured at the permissive temperature (20° C) during development and then shifted to the restrictive temperature (25° C) during adulthood. The *par-1* ts mutant shows significant lifespan extension compared to the wild-type N2 (Figure 1A and Supplementary Table 1). We further examined the temporal requirement of *par-1* in lifespan determination by RNAi knockdown at different stages. *par-1* RNAi knockdown only during development (Dev) was achieved by initiating the RNAi treatment upon embryonic hatching, and *par-1* RNAi was then shut down on Day 1 of adulthood by shifting animals to *dcr-1* RNAi, which abrogates the RNAi machinery [25]. Other animals were treated with the *par-1* RNAi either during adulthood only (AD) or throughout the whole life (Dev + AD). Compared to the control RNAi treatment, knockdown of *par-1* during development has no effect on lifespan, whereas *par-1* RNAi during adulthood or the whole life results in significantly prolonged longevity (Figure 1B and Supplementary Table 1). Therefore, adulthood inhibition of *par-1* is sufficient to extend lifespan.

To facilitate lifespan assays, low dose (20 μg / ml) of the DNA synthesis inhibitor 5-fluoro-2-deoxyuridine (FUdR) was supplemented to the worm culture to prevent progeny from hatching. It has been reported that FUdR, especially at high dose such as 100 μg / ml, has profound effects on *C. elegans* physiology and aging [26]. To examine whether the effect of *par-1* on lifespan is dependent on FUdR, we performed lifespan assays using the conditional infertility strain PX627, which shows auxin-inducible sterility and normal lifespan [27, 28]. Adult stage RNAi knockdown of *par-1* significantly extends lifespan in PX627 without the FUdR treatment (Supplementary Figure 1A). To test whether the *par-1* deficiency-induced lifespan extension is temperature-dependent, we performed lifespan assays at 20° C. *par-1* RNAi treated animals at this condition show significantly prolonged longevity similar to those at 25° C (Supplementary Figure 1B). Therefore, *par-1* is a bona fide regulator of lifespan.

### Inhibition of *par-1* significantly improves healthspan

Lifespan is one of the measurements of aging. Significantly prolonged longevity does not necessarily ensure the delay of aging. Healthspan assays, which include various stress resistance, human disease models and other age-related physiological studies, have been used to quantitatively assess the rate of aging. To further characterize the role of PAR-1 in aging, we treated animals with the control or *par-1* RNAi starting from the adulthood for two days, and then collected them for various healthspan measurements.

**Figure 2:**
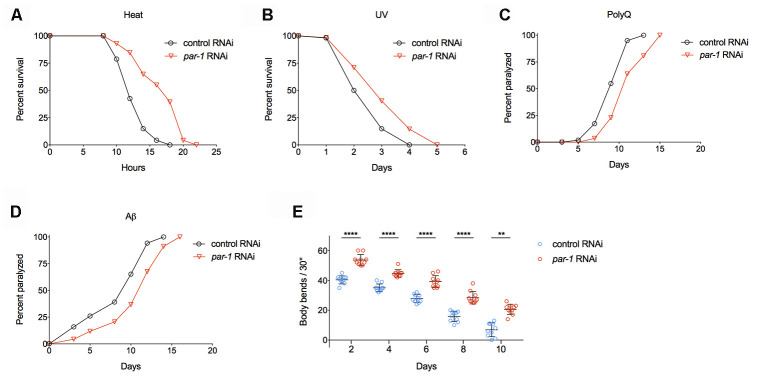
**RNAi knockdown of *par-1* significantly improves healthspan.** (**A**) Survival curves of wild-type animals treated with either the control or *par-1* RNAi at 35°C (*p* < 0.0001, log-rank test). (**B**) Survival curves of wild-type animals treated with either the control or *par-1* RNAi upon UV (2,000 J/m^2^) exposure (*p* = 0.0003, log-rank test). (**C**, **D**) Age-associated paralysis induced by body wall muscle expression of either Q35 (**C**) or Aβ (D) in animals treated with the control or *par-1* RNAi (*p* < 0.0001, log-rank tests). (**E**) Body bending rates of day 2, 4, 6, 8, and 10 adults treated with either the control or *par-1* RNAi (****, *p* < 0.0001, **, *p* = 0.0029, two-way ANOVA with Sidak's multiple comparison tests). Detailed quantitative data and statistical analyses are included in Supplementary Tables 2–5.

Previous studies have demonstrated that many long-lived mutants show intrinsic thermotolerance, and the increased ability to deal with stress often leads to lifespan extension [29–31]. We found RNAi knockdown of *par-1* significantly enhances thermotolerance (Figure 2A and Supplementary Table 2) and extends survival upon UV exposure (Figure 2B and Supplementary Table 3), suggesting *par-1* loss-of-function induced lifespan extension might be due to better somatic maintenance.

Proteotoxicity has been connected with many human degenerative diseases, such as Alzheimer’s, Parkinson’s, Huntington’s diseases and so on. Previously, researchers have constructed transgenic animals that express either polyQ (Q35) or human Aβ1-42 in *C. elegans* body wall muscles [32, 33]. These transgenic animals show age-dependent protein aggregations in muscle cells, which lead to proteotoxicity and eventually paralysis. Inhibition of *par-1* significantly delays age-associated, muscular proteotoxicity-induced paralysis in both the polyQ and Aβ models (Figure 2C, 2D and Supplementary Table 4).

Previous studies on the pathology of aging indicate that progressive muscular function decline serves as a reliable biomarker of aging in *C. elegans* [34]. Worm muscular function can be quantitatively measured by counting the numbers of body bends when animals are transferred into liquid. Animals with the *par-1* RNAi treatment show significantly enhanced mobility during aging compared to those treated with the control RNAi (Figure 2E and Supplementary Table 5).

To validate the role of *par-1* in healthspan, we tested the *par-1(zu310)* ts mutant for the thermotolerance, survival upon UV exposure and age-dependent muscular function decline two days after shifting to the restrictive temperature (25° C) since the late L4 stage. Compared to the wild-type N2, the *par-1(zu310)* ts mutant shows significantly increased thermotolerance (Supplementary Figure 2A), extended survival upon UV exposure (Supplementary Figure 2B), and better mobility during aging (Supplementary Figure 2C). Taken together, these results demonstrate that inhibition of *par-1* during adulthood significantly improves healthspan.

### PAR-1 mainly functions in the epidermis to regulate lifespan

**Figure 3:**
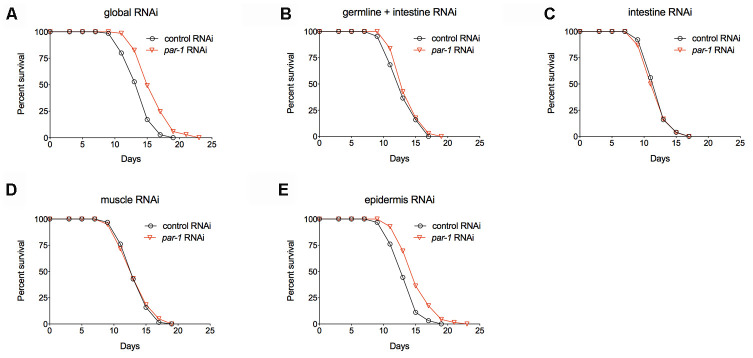
***par-1* mainly functions in the epidermis to regulate lifespan.** (**A**) Survival curves of animals treated with the global control or *par-1* RNAi (*p* < 0.0001, log-rank test). (**B**) Survival curves of animals treated with the germline plus intestine-enriched control or *par-1* RNAi (*p* = 0.1756, log-rank test). (**C**) Survival curves of animals treated with the intestine-enriched control or *par-1* RNAi (*p* = 0.6433, log-rank test). (**D**) Survival curves of animals treated with the muscle-enriched control or *par-1* RNAi (*p* = 0.7955, log-rank test). (**E**) Survival curves of animals treated with the epidermis-enriched control or *par-1* RNAi (*p* < 0.0001, log-rank test). In all cases, animals were treated with the control or *par-1* RNAi during the adulthood. Detailed quantitative data and statistical analyses are included in Supplementary Table 1.

In response to genetic and environmental influences, different tissues coordinately modulate physiology to affect aging at the whole organism level. To better understand the spatial requirement of PAR-1 in lifespan determination, we carried out tissue-enriched *par-1* RNAi knockdown and examined the effects on lifespan. RDE-1 is an Argonaute protein that is essential for the RNAi machinery as well as systemic RNAi [35]. Therefore, spatially restricted RNAi knockdown can be achieved by tissue-specific promoters driving *rde-1* transgenic rescue of *rde-1* loss-of-function mutations [36–39]. Mutations in *rrf-1*, which encodes an RNA-directed RNA polymerase, allow RNAi to be functional in both the germline and intestine [40, 41]. In contrast to the global *par-1* RNAi treatment (Figure 3A and Supplementary Table 1), tissue-enriched RNAi knockdown of *par-1* in the germline plus intestine, intestine or muscles does not affect lifespan (Figure 3B–3D and Supplementary Table 1), whereas the epidermis-enriched *par-1* RNAi treatment significantly extends lifespan (Figure 3E and Supplementary Table 1). Therefore, the epidermis is the main tissue in which PAR-1 functions to regulate lifespan.

### PAR-1 functions through the nutrient-responsive S6K-AMPK pathway to regulate lifespan

**Figure 4:**
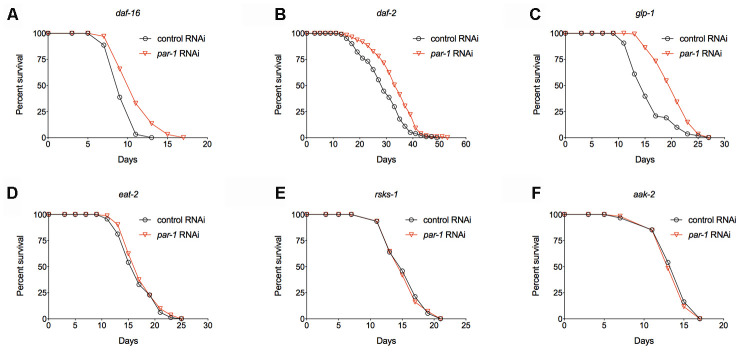
**Epistatic analysis of *par-1* for its effects on lifespan.** (**A**) Survival curves of the *daf-16* mutant treated with the control or *par-1* RNAi (*p* < 0.0001, log-rank test). (**B**) Survival curves of the *daf-2* mutant treated with the control or *par-1* RNAi (*p* < 0.0001, log-rank test). (**C**) Survival curves of the *glp-1* mutant treated with the control or *par-1* RNAi (*p* < 0.0001, log-rank test). (**D**) Survival curves of the *eat-2* mutant treated with the control or *par-1* RNAi (*p* = 0.1489, log-rank test). (**E**) Survival curves of the *rsks-1* mutant treated with the control or *par-1* RNAi (*p* = 0.7661, log-rank test). (**F**) Survival curves of the *aak-2* mutant treated with the control or *par-1* RNAi (*p* = 0.4647, log-rank test). In all cases, animals were treated with the control or *par-1* RNAi during the adulthood. Detailed quantitative data and statistical analyses are included in Supplementary Table 1.

In order to characterize the mechanisms of lifespan extension by *par-1* deficiency, we performed epistasis experiments to examine the genetic interactions between *par-1* and known longevity pathways. Mutations in DAF-2, the *C. elegans* ortholog of the insulin/IGF-1 receptor, more than double the adult lifespan that is dependent on the downstream DAF-16/FOXO transcription factor [4, 6, 7]. We found that *par-1* RNAi knockdown significantly extends lifespan of a *daf-16* null mutant (Figure 4A and Supplementary Table 1). Consistently, *daf-2* mutant animals treated with *par-1* RNAi show further lifespan extension compared to those treated with the control RNAi (Figure 4B and Supplementary Table 1). These results suggest that the mechanisms by which *par-1* functions to regulate lifespan are different from those by reduced insulin-like signaling.

Previous studies have demonstrated that germline-less animals produced by either laser ablation of the germline precursor cells or the *glp-1(e4144)* ts mutant show significantly prolonged longevity, and the underlying mechanisms involve cell non-autonomous activation of DAF-16 in the metabolic tissue [42, 43]. We found that *par-1* RNAi knockdown can further extend lifespan of the *glp-1(e4144)* ts mutant (Figure 4C and Supplementary Table 1). This result further supports that the *par-1* deficiency-induced lifespan extension is independent of DAF-16.

Dietary restriction (DR) is one of the most robust environmental manipulations that significantly delay aging. The *eat-2* mutant, which has impaired food intaking thus serves as a genetic mimic of DR, shows significant lifespan extension [44]. RNAi knockdown of *par-1* in the *eat-2* mutant background does not further extend lifespan (Figure 4D and Supplementary Table 1), suggesting overlapping mechanisms in the regulation of longevity. The nutrients-regulated TOR pathway also plays a key role in aging [12]. Inhibition of TOR or its downstream effector, the ribosomal S6 kinase, which is encoded by *rsks-1* in *C. elegans*, significantly extends lifespan [8, 9, 13]. Similar to the situation in the *eat-2* mutant background, inhibition of *par-1* does not affect lifespan of the long-lived *rsks-1* mutant (Figure 4E and Supplementary Table 1). It has been shown that the prolonged longevity of the *rsks-1* mutant requires AAK-2, the catalytic subunit of the key energy homeostasis regulator AMPK [45]. The *par-1* deficiency induced longevity can be completely suppressed by the *aak-2* null mutant (Figure 4F and Supplementary Table 1). Therefore, *par-1* functions through nutrients-responsive mechanisms to regulate lifespan.

### Knockdown of *par-1* activates AMPK and reduces lipid accumulation in the metabolic tissue

**Figure 5:**
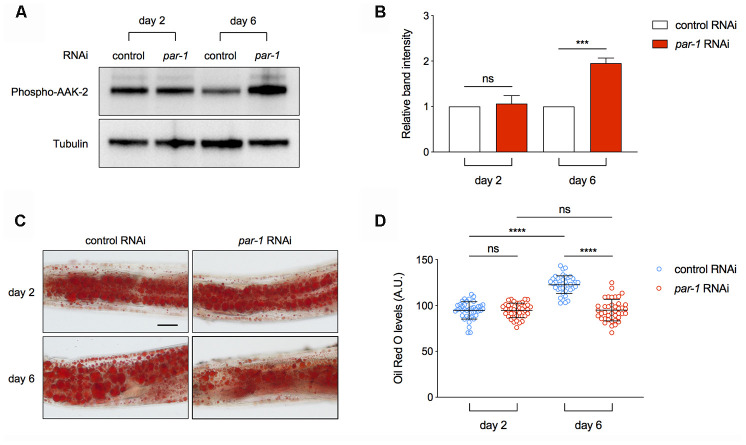
**Inhibition of *par-1* activates AMPK and decreases lipid levels in the metabolic tissue.** (**A**, **B**) Immunoblots (**A**) and quantification (**B**) of phospho-AAK-2 (AMPKα) and tubulin using proteins extracted from dissected intestinal tissues of day 2 and day 6 adult animals treated with the control or *par-1* RNAi. Ratio of the phospho-AAK-2 band intensity to that of tubulin was normalized to the control RNAi treated samples. Data are represented as mean ± SD based on three independent biological replicates. (**C**, **D**) Representative Oil Red O staining images (**C**) and quantification (**D**) of the staining signal in day 2 and day 6 adult animals treated with the control or *par-1* RNAi. Data are represented as mean ± SD. ns, not significant, ****, *p* < 0.0001 (n = 40, two-way ANOVA with Sidak's multiple comparisons tests). Scale bar, 50 μm.

AMPK serves as a key energy homeostasis regulator to promote catabolism under starved conditions. It contains three subunits, and phosphorylation of a highly conserved threonine (T172) on the α subunit is required for its kinase activity [46]. The *rsks-1* mutant shows increased levels of phospho-AAK-2 [45]. To access whether *par-1* RNAi can activate AAK-2 especially in the metabolic tissue, we performed immuno-blots to determine age-associated changes in phospho-AAK-2 levels using micro-dissected intestine, which is the major metabolic tissue in *C. elegans*. *par-1* RNAi knockdown leads to increased phospho-AAK-2 levels in Day 6 adult animals compared to the control RNAi treatment (Figure 5A, 5B). The age-dependent activation of AMPK by *par-1* loss-of-function was also confirmed in the *par-1(zu310)* ts mutant (Supplementary Figure 3A, 3B).

One important function of AMPK is to promote lipid breakdown for more energy production. In order to determine whether *par-1* RNAi-induced AMPK activation has any physiological consequence, we performed Oil Red O straining and quantification to estimate triglyceride levels in the intestine of control or *par-1* RNAi treated animals at different ages. The control RNAi treated animals show age-associated increase in triglycerides, whereas this change is abrogated by *par-1* RNAi knockdown (Figure 5C, 5D). This age-dependent increase in lipid droplets can also be suppressed by the *par-1(zu310)* ts mutant (Supplementary Figure 3C, 3D). Therefore, inhibition of *par-1* during adulthood leads to age-dependent AMPK activation and improved lipid homeostasis.

## DISCUSSION

Aging can be significantly affected by genetic or environmental factors. Many key modulators of aging, such as the IGF-1 receptor and TOR, also play important roles in cellular energy homeostasis, growth and reproduction [2]. Knock-out mutants of these genes show lethality, whereas inhibition of them via hypomorphic mutations or RNAi knockdown significantly extends lifespan. Studies on wild-derived *C. elegans* showed that there is a negative correlation between longevity and developmental rates [47]. Taken together, these findings suggest that there are potential links between development and aging.

Genome-wide RNAi screens have been performed to identify lifespan determinants in *C. elegans* [16, 17]. Animals were subject to various RNAi treatments during the whole life. Thus, essential genes were likely to be excluded from the screens due to the lethality caused by RNAi knockdown during development. Subsequent studies on essential genes via RNAi knockdown only during adulthood demonstrate that these genes are enriched with negative modulators of aging [18, 19]. This is consistent with the antagonistic pleiotropy theory of aging, which suggests that genes essential for growth and development are likely to modulate aging later in life [48]. In this study, we focused on *par-1*, a conserved kinase gene that serves as a key cellular polarity regulator during the embryonic development [22, 23], for its roles in adulthood. Using a conditional loss-of-function mutant and RNAi knockdown, we found that inhibition of *par-1* during adulthood is sufficient to delay aging, as indicated by not only significantly prolonged longevity, but also significantly improved healthspan. These results further support the notion that developmentally essential genes are likely to function as key modulators of aging later in life.

It has been well documented that different tissues function coordinately to modulate aging in multicellular organisms. Many key regulators of lifespan, such as the DAF-16/FOXO transcription factor, functions in the intestine to affect longevity and aging-related phenotypes [49–51]. Using tissue-enriched RNAi strains, we find that inhibition of *par-1* in the epidermis, but not other tissues, leads to significantly prolonged longevity. The epidermal tissue contains several cell types that are involved in protection, innate immunity, metabolism, proteostasis and so on [52]. A previous study demonstrates that reduced insulin-like signaling delays aging via the SKN-1 transcription factor-mediated activation of collagens and other extracellular matrix genes, and the delay of age-associated collagens expression decline is a general protective mechanism for other anti-aging interventions [53]. It has been shown that PAR-1 functions in epithelial cells to regulate the morphogenesis of vulva during larval development. Although PAR-1 shows asymmetric distribution, there were no detectable changes in various epithelial polarity markers in this process [23]. Therefore, it will be interesting to determine in which epidermal cell types PAR-1 functions to regulate lifespan and whether PAR-1 affects aging by regulating cellular polarity in these cells in future studies.

AMPK serves as the key regulator of energy homeostasis. When the nutrient levels are low, a highly conserved threonine residue (T172) on the AMPK catalytic α subunit will be phosphorylated by upstream kinases such as PAR-4/LKB1 [45, 54] and VRK-1 [55] to ensure the activation of its kinase activity. AMPK helps to restore cellular energy homeostasis by activating catabolism and inhibiting energy costly biological processes [46]. AMPK has been shown to be responsible for the prolonged longevity by mutations in *daf-2*, *rsks-1*, perturbation of mitochondria and certain forms of dietary restriction [45, 56–58]. A recent study showed that simultaneous inhibition of the IGF-1 and TOR pathways reduces cytochrome c production in the germline, which leads to cell non-autonomous activation of the mitochondrial stress response and AMPK in the intestine to ensure lifespan extension [15]. In this study, we find AMPK is required for *par-1* RNAi knockdown induced lifespan extension, and inhibition of *par-1* causes age-dependent activation of AMPK in the intestine. These findings highlight the important role of AMPK in the metabolic tissue in aging. Serving as a S/T protein kinase, PAR-1 is unlikely to be a direct upstream regulator of AMPK since inhibition of *par-1* leads to increased AMPKα phosphorylation. Similar phenotype was also reported in the knockout mutant of RSKS-1/ribosomal S6 kinase [14, 45]. Further characterization of the underlying mechanisms will help to better understand the network of aging modulators.

Lipid metabolism plays important but complicated roles in health and aging. Obesity has been connected with multiple pathologies. Intriguingly, many long-lived *C. elegans* mutants, such as *daf-2* (IGF-1 receptor), *rsks-1* (ribosomal S6 kinase), germline-less *glp-1* (Notch), show significantly increased lipid accumulation [5, 59, 60]. Further studies demonstrate that there is no simple correlation between lipid levels and aging, whereas effects of lipid metabolism on aging involve more specific mechanisms [61]. The long-lived germline-less *glp-1* mutant has increased expression of the lysosomal lipase LIPL-4, which promotes the production of certain lipid species such as oleoylthanolamide to activate nuclear hormone receptors NHR-49 and NHR-80 for prolonged longevity [62, 63]. A H3K4me3 methyltransferase mutant shows intestinal up-regulation of Δ-9 fatty acid desaturases to promote mono-unsaturated fatty acids production and lifespan extension [10]. Here we find that worms show age-dependent increase in neutral lipid accumulation in the intestine, whereas inhibition of *par-1* suppresses this phenotype. Since *par-1* RNAi treatment also activates AMPK in a similar spatiotemporal manner, and AMPK promotes lipids breakdown, we speculate that *par-1* knockdown-induced AMPK activation is responsible for the anti-aging effect via modulating lipid homeostasis. Studies on fatty acid profiles among strains of different lifespan showed that fatty acid chain length and the susceptibility to oxidation negatively correlate with longevity [64]. Therefore, it will be interesting to characterize the changes in lipid profile upon *par-1* inhibition, and to determine whether these changes contribute to the anti-aging effect in future studies.

In summary, we identify PAR-1 as a novel modulator of aging in a spatiotemporal specific manner. Genetic and molecular analyses reveal that PAR-1 functions in the nutrient-responsive S6K-AMPK pathway to determine lifespan via regulating age-dependent AMPK activation and lipid metabolism. Further studies on PAR-1 for its molecular role in lifespan and healthspan will help to better understand the intrinsic links among development, metabolism and aging.

## MATERIALS AND METHODS

### *C. elegans* strains and maintenance

Worms were cultured on the NGM (nematode growth media) agar plates seeded with *E. coli* OP50 at 20° C unless otherwise stated. The following *C. elegans* strains were obtained from the Caenorhabditis Genome Center: Bristol N2 as the wild-type strain, AM140 *rmIs132[P(unc-54) Q35::YFP] I*, CB1370 *daf-2(e1370) III*, CF1038 *daf-16(mu86) I*, CB4037 *glp-1(e2144) III*, CL2006 *dvIs2[pCL12(unc54/human Abeta peptide 1-42 minigene) + pRF4]*, DA465 *eat-2(ad465) II*, KK822 *par-1(zu310) V*, MAH23 *rrf-1(pk1417) I*, NR222 *rde-1(ne219) V; kzIs9[pKK1260(lin-26p::nls::GFP) + pKK1253(lin-26p::rde-1) + pRF6(rol-6(su1006)]*, PX627 *fxIs1 [pie-1p::TIR1::mRuby] I; spe-44(fx110 [spe-44::degron]) IV*, RB1206 *rsks-1(ok1255) III*, RB754 *aak-2(ok524) X*, VP303 *rde-1(ne219) V; kbIs7[Pnhx-2::rde-1 + rol-6]*, WM118 *rde-1(ne300) V; neIs9[myo-3::HA::RDE-1 + pRF4(rol-6(su1006))] X*. The following strain was generated in Di Chen lab: DCL4 *rsks-1(ok1255) III*. The following strain was generated in Pankaj Kapahi lab: XA8205 *aak-2(ok524) X*.

### RNAi by feeding

RNAi experiments were performed by feeding worms *E. coli* strain HT115 (DE3) transformed with either the empty vector L4440 as the control or gene-targeting constructs from the Ahringer RNAi Collection. All RNAi clones were verified by DNA sequencing. Overnight bacterial culture was seeded onto NGM plates supplemented with IPTG (1 mM) and Ampicillin (100 μg / ml) and incubated at room temperature overnight to induce the production of double-stranded RNAs. For *par-1* RNAi knockdown during development, gravid adult worms were allowed to lay eggs on *par-1* RNAi plates at 20° C for 2 hours before removed. The progeny at late L4 stages was transferred onto *dcr-1* RNAi plates with 20 μg / ml (+)-5-fluorodeoxyuridine (FUdR) and incubated at 25° C for two days before moved onto control RNAi plates. For *par-1* RNAi knockdown during adulthood, late L4 larvae were transferred onto *par-1* RNAi plates with FUdR (20 μg / ml) and incubated at 25° C for various assays.

### Lifespan assays

Worms at the late L4 stages were transferred to fresh NGM or RNAi plates and incubated at 25° C or 20° C for survival assays. FUdR (20 μg / ml) was used during day 1 to day 7 of adulthood to prevent progeny production. Animals were scored as alive, dead (no response to gentle touch) or lost (death from non-ageing causes such as sticking to the plate walls, internal hatching or bursting in the vulval region) every other day. Survival curves were plotted with the GraphPad Prism software and statistical analyses were performed using the log-rank method.

### Thermotolerance assays

Synchronized L4 larvae were transferred onto the control or *par-1* RNAi plates and incubated at 25° C for two days. Adult animals were then incubated at 35° C for survival assays. Animals were scored as alive or dead every other hour.

### UV stress assays

Synchronized L4 larvae were transferred onto the control or *par-1* RNAi plates and incubated at 25° C for two days. Adult animals were transferred to empty NGM plates and exposed to UV radiation (2,000 J / m^2^). 50 μl of the OP50 bacterial culture was then added to each plate and animals were monitored for survival daily.

### Proteotoxicity-induced paralysis assays

Synchronized AM140 (Poly Q) or CL2006 (Aβ) L4 larvae were transferred onto the control or *par-1* RNAi plates and incubated at 25° C. Animals were monitored for paralysis, which is defined as no forward movement upon gentle touch with the platinum wire, every other day. Paralysis curves were plotted with the GraphPad Prism software and statistical analyses were performed using the log-rank method.

### Age-dependent muscular function decline assays

Adult animals that have been treated with either the control or *par-1* RNAi since the late L4 larval stage were individually transferred into the S buffer (100 mM NaCl and 50 mM potassium phosphate [pH 6.0]) and let to rest for 1 minute before the numbers of body bending per 30 seconds were counted. Scatter graphs were plotted with the GraphPad Prism software and evaluated using two-way ANOVA with Sidak's multiple comparison tests.

### Worm intestine micro-dissection

Day 2 or day 6 adult animals treated with either the control or *par-1* RNAi since the late L4 stage were transferred into the S buffer on a glass slide. Heads of animals were cut off near the pharynx using syringe needles to collect the intestinal tissue. At least 100 intestine tissues were collected in the protein extraction buffer (150 mM NaCl, 1 mM EDTA, 0.25% SDS, 1.0% NP-40, 50 mM Tris-HCl [pH7.4], Roche complete protease inhibitors and phosSTOP phosphatase inhibitors) for each sample.

### Western blots and antibodies

Approximately equal amount of dissected intestinal tissues was collected into the protein extraction buffer supplemented with the 4 × SDS loading buffer. Samples were boiled for 10 minutes before resolving on precast SDS-PAGE gels (GenScript). Antibodies used in Western blots include anti-Phospho-AMPKα (CST, 2535S) and monoclonal anti-Tubulin Alpha (Sigma, T6074).

### Lipid staining by Oil Red O

Day 2 or day 6 adult animals treated with either the control or *par-1* RNAi since the late L4 stage were collected and fixed in 1% formaldehyde and frozen at -80° C. The samples were subject to three cycles of freezing and thawing with dry ice / ethanol bath and a stream of warm water, respectively. After washed twice with the S buffer, animals were incubated in the Oil red O (3 mg / ml) solution for 30 minutes at the room temperature. Animals were then washed with the S buffer and incubated on ice for 15 min. Images were taken using a Nikon Eclipse Ni-U microscope equipped with a DS-Fi2 color CCD. Mean intensity of Oil Red O signal in the second pair of intestinal cells was quantified using the Image J software.

### Statistical analysis

Survival curves and scatter graphs were plotted with the GraphPad Prism software. Survival curves were evaluated with log-rank tests. Data in scatter graphs were plotted as means ± SD and evaluated using two-way ANOVA or t -tests. *p* < 0.05 was considered significant.

## Supplementary Material

Supplementary Figures

Supplementary Tables
