# Supplementary material for: Inhibition of PAR-1 delays aging via activating AMPK in C. elegans
Source: Aging (Albany NY). 2020 Nov 20;12(24):25700–17. doi: 10.18632/aging.104180 (PMC7803586; doi:10.18632/aging.104180)
Supplement: Supplementary Figures [file aging-12-104180-s001.pdf]

SUPPLEMENTARY FIGURES

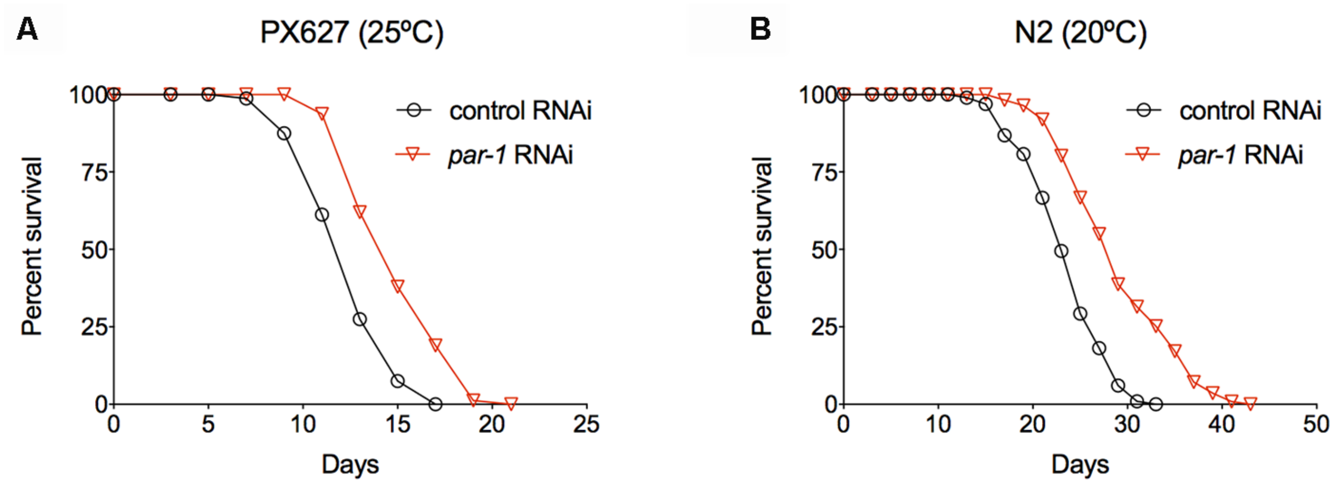

Supplementary Figure 1. Lifespan extension induced by *par-1* RNAi knockdown is not dependent on FUdR or temperature. (**A**) Survival curves of the auxin-inducible infertility strain PX627 treated with either the control or *par-1* RNAi during adulthood at 25º C. The *par-1* RNAi treatment significantly extends lifespan by 21% ($p < 0.0001$, log-rank test). (**B**) Survival curves of the wild-type N2 treated with either the control or *par-1* RNAi during adulthood at 20º C. The *par-1* RNAi treatment significantly extends lifespan by 23% ($p < 0.0001$, log-rank test).

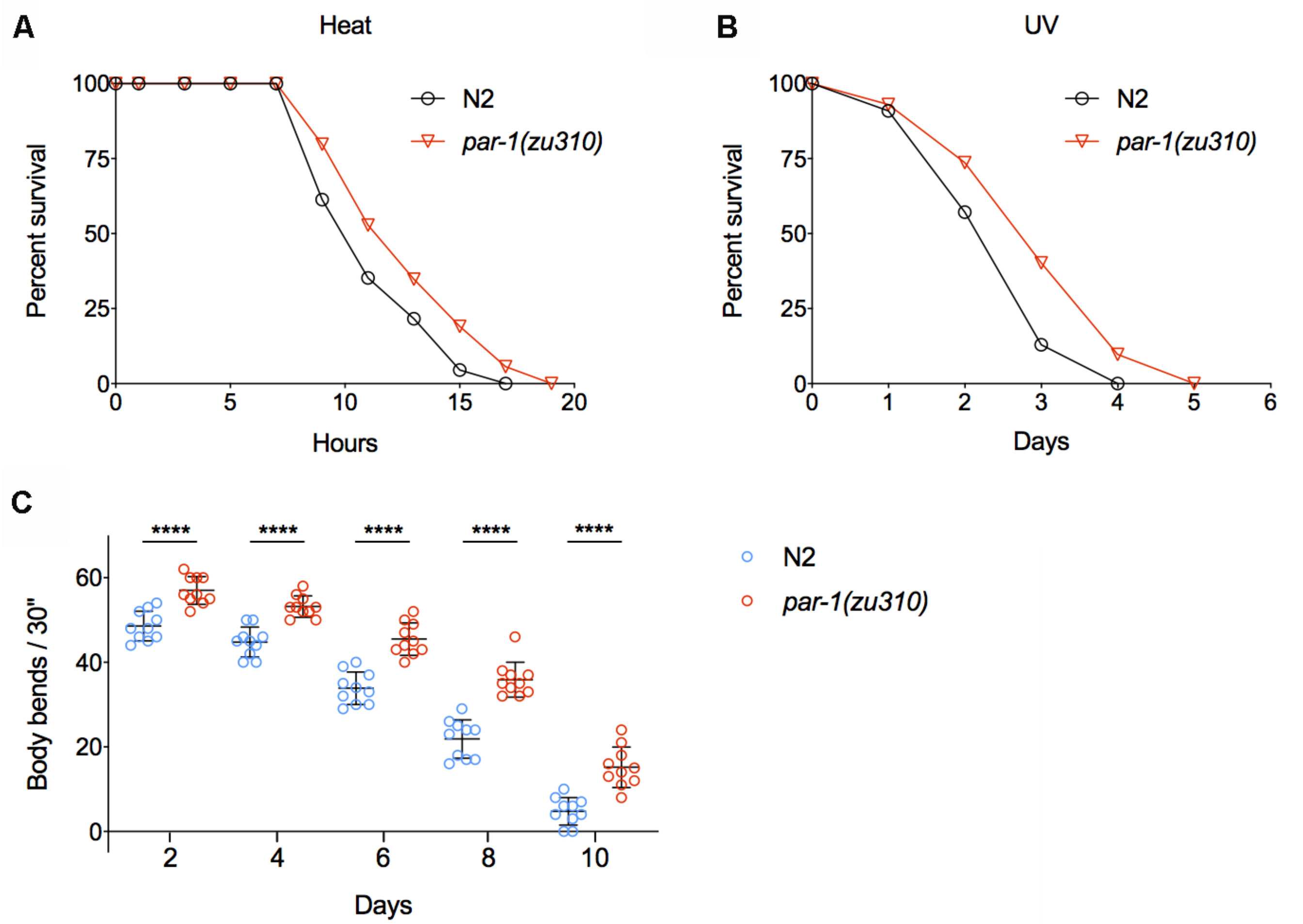

Supplementary Figure 2. The *par-1(zu310)* mutant shows significantly improved healthspan. (**A**) Survival curves of the wild-type N2 and *par-1(zu310)* animals at 35°C (p = 0.0007, log-rank test). (**B**) Survival curves of the wild-type N2 and *par-1(zu310)* animals upon UV (2,000 J/m^2) exposure (p = 0.0001, log-rank test). (**C**) Body bending rates of N2 and *par-1(zu310)* on day 2, 4, 6, 8, and 10 of adulthood (****, $p < 0.0001$, two-way ANOVA with Sidak's multiple comparison tests).

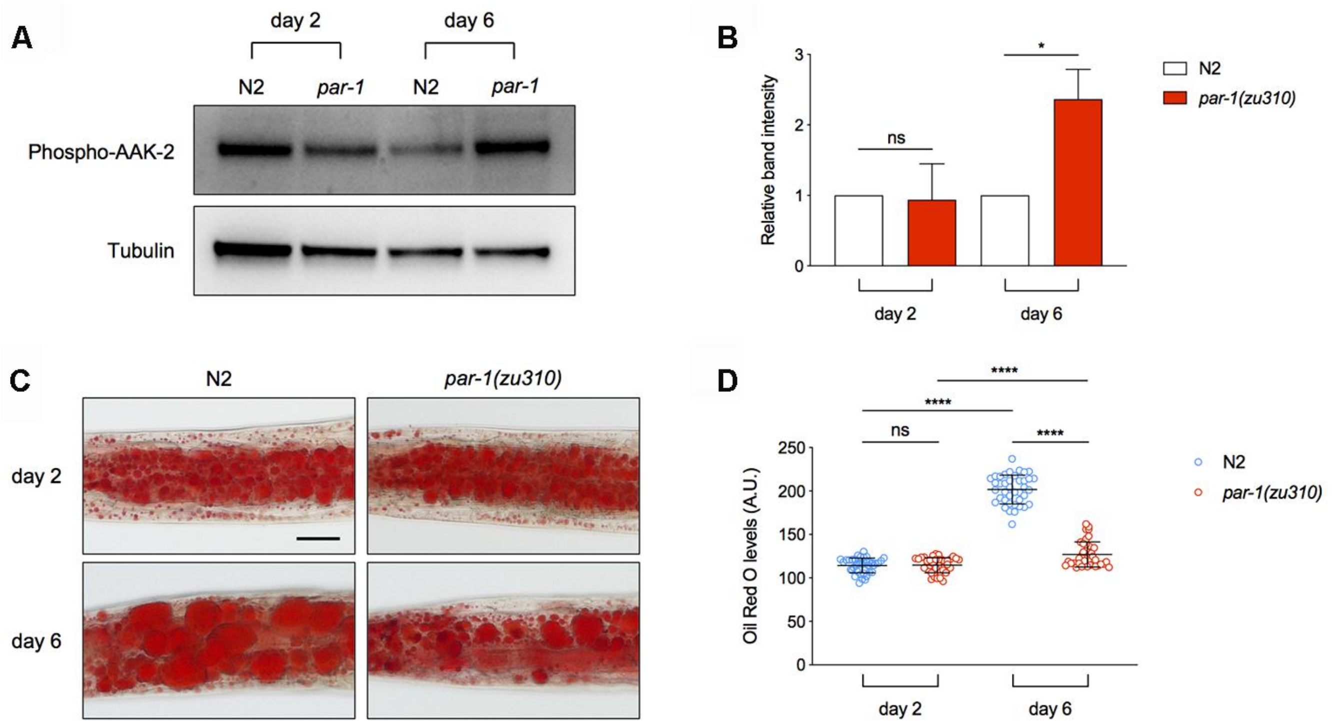

Supplementary Figure 3. The *par-1(zu310)* mutant shows AMPK activation and decreased lipid levels in the metabolic tissue. (**A**, **B**) Immunoblots (**A**) and quantification (**B**) of phospho-AAK-2 and tubulin using proteins extracted from dissected intestinal tissues of the wild-type N2 and *par-1(zu310)* mutant animals. Ratio of the phospho-AAK-2 band intensity to that of tubulin was normalized to the wild-type N2 animals. Data are represented as mean ± SD based on two independent biological replicates. ns, not significant, *, $p = 0.0443$ (unpaired t -tests). (**C**, **D**) Representative Oil Red O staining images (**C**) and quantification (**D**) of the staining signal in the wild-type N2 and *par-1(zu310)* mutant day 2 and day 6 adult animals. Data are represented as mean ± SD. ns, not significant, ****, $p < 0.0001$ (n = 40, two-way ANOVA with Sidak's multiple comparisons tests). Scale bar, 50 μm.
