# Supplementary material for: Inhibition of PAR-1 delays aging via activating AMPK in C. elegans
Source: Aging (Albany NY). 2020 Nov 20;12(24):25700–17. doi: 10.18632/aging.104180 (PMC7803586; doi:10.18632/aging.104180)
Supplement: Supplementary Tables [file aging-12-104180-s002.pdf]

SUPPLEMENTARY TABLES

Supplementary Table 1. Statistical analyses of lifespan assays.

Genotype	RNAi	Tissue [a]	Lifespan (days)		Percent of the control [b]	n [c]	*p* [d]
			Mean	Max			
Effect of the *par-1(zu310)* mutant on lifespan (Figure 1A)							
N2	/	/	13.66, 11.71	19, 17	/	79, 56	/
par-1(zu310)	/	/	16.39, 14.53	25, 19	120%, 124%	49, 68	<0.0001, <0.0001
Temporal requirement of *par-1* in lifespan regulation (Figure 1B)							
N2	control	global	13.93, 13.14	19, 17	/	84, 58	/
	par-1 (Dev)		14.23, 12.88	19, 17	102%, 98%	60, 50	0.5368, 0.3680
	par-1 (AD)		17.18, 14.69	23, 19	123%, 112%	80, 71	<0.0001, <0.0001
	par-1 (Dev+AD)		17.73, 14.74	25, 21	127%, 112%	74, 69	<0.0001, <0.0001
Spatio requirement of *par-1* in lifespan regulation (Figure 3)							
N2	control	global	14.03, 13.63	19, 17	/	70, 63	/
	par-1		16.28, 15.79	23, 21	116%, 116%	69, 66	<0.0001, <0.0001
rrf-1	control	germline + intestine	13.32, 13.51	17, 17	/	63, 55	/
	par-1		13.94, 13.76	19, 17	105%, 102%	68, 58	0.1756, 0.2838
rde-1; kbIs7	control	intestine	12.36, 12.05	17, 15	/	50, 59	/
	par-1		12.15, 11.97	17, 15	98%, 99%	54, 60	0.6433, 0.6665
rde-1; neIs9	control	muscle	13.67, 13.16	19, 17	/	63, 61	/
	par-1		13.67, 13.44	19, 17	100%, 102%	60, 64	0.7955, 0.6097
rde-1; kzIs9	control	epidermis	13.63, 13.44	19, 17	/	63, 63	/
	par-1		15.43, 16.21	23, 21	113%, 121%	69, 63	<0.0001, <0.0001
Epistatic analysis of *par-1* for its effect on lifespan (Figure 4)							
daf-16	control	global	9.61, 11.40	13, 17	/	62, 177	/
	par-1		11.24, 13.06	17, 21	117%, 115%	67, 174	<0.0001, <0.0001
daf-2	control	global	28.82, 29.99	49, 39	/	101, 75	/
	par-1		33.46, 33.64	53, 41	116%, 112%	109, 84	<0.0001, <0.0001
glp-1	control	global	15.24, 17.14	27, 23	/	139, 58	/
	par-1		20.30, 18.97	27, 25	133%, 111%	123, 61	<0.0001, 0.0089
eat-2	control	global	16.77, 15.75	25, 21	/	192, 69	/
	par-1		17.20, 17.36	25, 21	103%, 110%	131, 67	0.1489, 0.0053
rsks-1	control	global	15.53, 14.94	21, 21	/	60, 62	/
	par-1		15.39, 14.78	21, 21	99%, 99%	61, 64	0.7661, 0.7359
aak-2	control	global	12.05, 12.30	15, 17	/	61, 60	/
	par-1		11.87, 12.51	15, 17	99%, 102%	60, 61	0.4647, 0.6463

[a], tissues in which RNAi is mainly effective.
[b], changes in mean lifespan compared to the control.
[c], numbers of animals scored.
[d], log-rank tests.

Supplementary Table 2. Statistical analyses of thermotolerance assays.

Genotype	RNAi	Survival (hours)		Percent of the control [a]	n [b]	*p* [c]
		Mean	Max			
N2	control	15.31, 12.81	20, 18	/	61, 47	/
	par-1	18.11, 15.70	22, 20	118%, 123%	57, 54	<0.0001, <0.0001

[a], changes in the mean survival compared to the control.
[b], numbers of animals scored.
[c], log-rank tests.

Supplementary Table 3. Statistical analyses of UV stress assays.

Genotype	RNAi	Survival (days)		Percent of the control [a]	n [b]	*p* [c]
		Mean	Max			
N2	control	2.63, 2.90	4, 4	/	54, 154	/
	par-1	3.24, 3.39	5, 6	123%, 117%	59, 160	0.0003, <0.0001

[a], changes in the mean survival compared to the control.
[b], numbers of animals scored.
[c], log-rank tests.

Supplementary Table 4. Statistical analyses of proteotoxicity assays.

Genotype	RNAi	Paralysis (days)		Percent of the control [a]	n [b]	*p* [c]
		Mean	Max			
polyQ-induced paralysis						
rmIs132(unc-54p::Q35::YFP)	control	9.48, 10.82	13, 15	/	42, 113	/
	par-1	11.52, 12.40	15, 17	122%, 115%	42, 100	<0.0001, <0.0001
Aβ-induced paralysis						
dvIs2(unc-54p::Aβ1-42 + pRF4)	control	8.93, 10.52	14, 13	/	69, 63	/
	par-1	11.24, 11.92	16, 15	126%, 113%	68, 61	<0.0001, <0.0001

[a], changes in the mean paralysis time compared to the control.
[b], numbers of animals scored.
[c], log-rank tests.

Supplementary Table 5. Statistical analyses of muscular function assays.

Age	RNAi	Number of body bends / 30"	Average	STD	n [a]	*p* [b]
day 2	control	39, 35, 38, 42, 40, 41, 42, 40, 42, 45	40.4	2.72	10	/
	par-1	54, 52, 60, 50, 54, 50, 51, 52, 54, 60	53.7	3.65	10	<0.0001
day 4	control	39, 35, 32, 33, 35, 33, 34, 35, 40, 35	35.1	2.56	10	/
	par-1	44, 45, 51, 45, 43, 46, 42, 43, 45, 43	44.7	2.54	10	<0.0001
day 6	control	31, 26, 25, 26, 27, 32, 28, 30, 24, 30	27.9	2.73	10	/
	par-1	39, 40, 45, 35, 41, 36, 39, 35, 36, 46	39.2	3.94	10	<0.0001
day 8	control	12, 20, 19, 14, 10, 16, 13, 18, 19, 18	15.9	3.45	10	/
	par-1	26, 27, 25, 25, 30, 26, 33, 28, 38, 26	20.6	4.20	10	<0.0001
day 10	control	5, 1, 11, 0, 8, 11, 11, 2, 13, 6	6.9	4.61	10	/
	par-1	22, 26, 17, 20, 23, 23, 22, 20, 14, 19	20.6	3.41	10	0.0029

[a], numbers of animals scored.
[b], two-way ANOVA with Sidak's multiple comparison tests.
